# Imaging manifestations of von Hippel-Lindau disease: an illustrated guide focusing on the central nervous system

**DOI:** 10.1590/0100-3984.2021.0080-en

**Published:** 2022

**Authors:** João Luiz Veloso Mourão, Luiz Fernando Monte Borella, Juliana Ávila Duarte, Mariana Dalaqua, Daniel Alvarenga Fernandes, Fabiano Reis

**Affiliations:** 1 Department of Radiology, Universidade Estadual de Campinas (Unicamp), Campinas, SP, Brazil.; 2 Department of Radiology and Diagnostic Imaging, Hospital de Clínicas de Porto Alegre (HCPA), Porto Alegre, RS, Brazil.; 3 Department of Radiology, Hôpitaux Universitaires de Genève, Geneva, Switzerland.

**Keywords:** von Hippel-Lindau disease, Hemangioblastoma, Endolymphatic sac/pathology, Magnetic resonance imaging, Tomography, X-ray computed, Doença de von Hippel-Lindau, Hemangioblastoma, Saco endolinfático/patologia, Ressonância magnética, Tomografia computadorizada

## Abstract

Von Hippel-Lindau (VHL) disease is a rare, autosomal dominant inherited syndrome that affects the germline of the VHL gene, a tumor suppressor gene. VHL disease is characterized by the multisystemic development of a variety of benign and malignant tumors, especially in the central nervous system (CNS). Such tumors include retinal and CNS hemangioblastomas, as well as endolymphatic sac tumors. The various tumor sites are responsible for the diversity of signs and symptoms related to the disease. The mean age at symptom onset is 33 years. Despite medical advances, the average life expectancy of patients with VHL disease is 49 years. Imaging plays a pivotal role in the clinical diagnosis and is essential to the follow-up of patients with VHL disease. This pictorial essay describes characteristic CNS manifestations of VHL disease-related tumors that all radiology residents should be aware of.

## Abstract (pt)

A doença de von Hippel-Lindau (VHL) é uma síndrome hereditária autossômica dominante rara que afeta a linha germinativa do gene VHL, um gene supressor tumoral. A doença de VHL é caracterizada pelo desenvolvimento multissistêmico de uma variedade de tumores benignos e malignos, especialmente no sistema nervoso central (SNC). Dentre eles, destacam-se hemangioblastomas retinianos e do SNC, e o tumor do saco endolinfático. Os diferentes locais dos tumores justificam a diversidade de sinais e sintomas relacionados à doença, que usualmente se manifestam com a idade média de 33 anos. Apesar dos avanços da medicina, a expectativa de vida média desses pacientes é de 49 anos. Exames de imagem têm papel fundamental no diagnóstico e são essenciais no seguimento dos pacientes com doença de VHL. Este ensaio iconográfico descreve as manifestações características dos tumores do SNC relacionados à doença de VHL que todos os residentes de radiologia devem saber.

## INTRODUCTION

Von Hippel-Lindau (VHL) disease is an autosomal dominant hereditary disease that is characterized by the formation of tumors. The disease is related to germline mutations in the VHL gene, which acts as a tumor suppressor gene and is located on the short arm of chromosome 3, at locus 3p25-26^(1,2)^. The estimated incidence of VHL disease is 1:36,000 population, with penetrance > 90% as of 65 years of age^(3)^.

The VHL disease typically manifests in young adulthood, with an average age at onset of 33 years, and predisposes affected patients to the development of benign and malignant tumors, mainly in the central nervous system (CNS) and viscera^(4)^. The average life expectancy of patients with VHL disease is 49 years, the most common causes of death being clear cell renal carcinoma and hemangioblastoma^(5)^. Most of the lesions related to the disease are treatable, and, according to various protocols, monitoring is recommended.

VHL disease can be classified by clinical phenotype, each of which correlates with a specific genotype^(1)^: type 1—low risk for pheochromocytoma and high risk for hemangioblastomas, clear cell renal carcinoma, cysts, and pancreatic neuroendocrine tumors; type 2A—high risk for pheochromocytoma and low risk for clear cell renal carcinoma; type 2B—high risk for pheochromocytoma and clear cell renal carcinoma; and type 2C—high risk only for pheochromocytoma. Characteristic CNS tumors in VHL disease include retinal, cerebellar, and spinal cord hemangioblastomas, as well as endolymphatic sac tumors^(3)^.

A clinical diagnosis of VHL disease can be considered under the following circumstances: in a patient with a family history of VHL disease and at least one of the tumors characteristic of the disease (retinal hemangioblastoma, CNS hemangioblastoma, clear cell renal carcinoma, pancreatic neuroendocrine tumor, or endolymphatic sac tumor); in a patient with two or more retinal or CNS hemangioblastomas; in a patient with a retinal or CNS hemangioblastoma, plus at least one of the visceral tumors characteristic of VHL disease, excluding renal and epididymal cysts^(1)^. Genetic testing for germline mutations in the VHL gene can also confirm the diagnosis. In this context, imaging examinations play an important role in the diagnosis and follow-up of patients with VHL disease.

## INTRACRANIAL MANIFESTATIONS

VHL disease is characterized by CNS hemangioblastomas, which affect 60-80% of patients with the disease. Hemangioblastomas are benign tumors (classified as grade I tumors by the World Health Organization) that are multifocal, being characterized histologically by a large vascular network and vacuolated stromal cells, which can be quite voluminous. Hemangioblastomas can present as nodular or solid/cystic lesions^(3)^, affecting mainly the cerebellum and spinal cord^(6)^. The evolution of such lesions typically includes phases of growth and phases of stability, the average age at the onset of symptoms being 33 years. The symptoms vary depending on the expansile effect and the tumor site^(1,3)^.

## CEREBELLAR HEMANGIOBLASTOMAS

**Figure 1:**
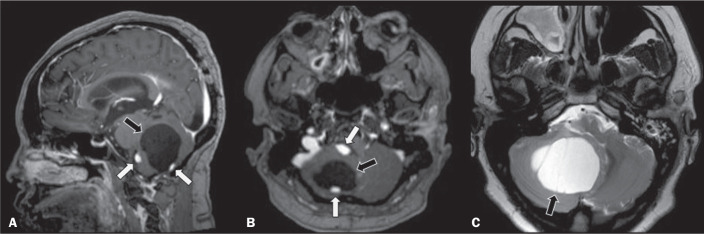
A 30-year-old female patient with VHL disease and cerebellar hemangioblastomas. Contrast-enhanced sagittal and axial T1-weighted MRI sequences (A and B, respectively), showing solid/cystic lesions with an expansile effect in the right cerebellar hemisphere, in which the cystic component presents a hypointense signal (black arrows) and the eccentric solid nodules (white arrows) show intense enhancement, the two features collectively resulting in compression of the fourth ventricle and contralateral deviation of the vermis. Axial T2-weighted MRI sequence (C) showing the cystic component, with a hyperintense signal (arrow), compressing the medulla oblongata and causing it to rotate slightly in a clockwise direction.

**Figure 2:**
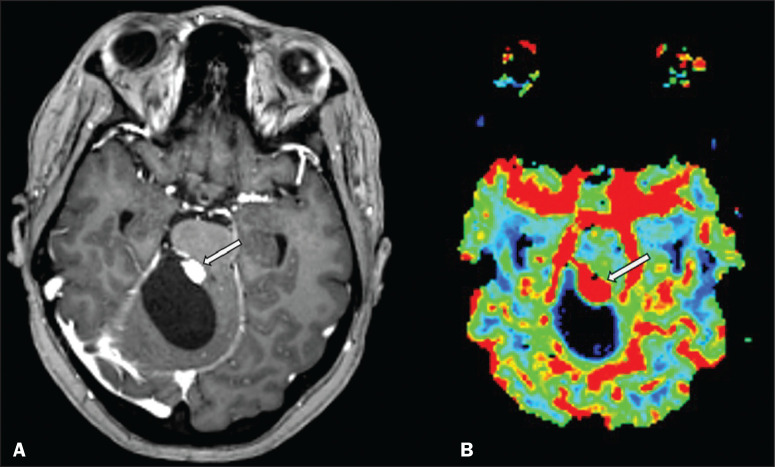
A 30-year-old female patient with VHL disease and cerebellar hemangioblastomas. Contrast-enhanced axial T1-weighted MRI sequence (A) showing a solid/cystic lesion in the right cerebellar hemisphere, characterized by the hypointense signal of its cystic component and the intense enhancement of its solid eccentric component (arrow). The cerebral blood volume map (B) demonstrates an increase in perfusion in the region with contrast enhancement (arrow).

Among individuals with VHL disease, the reported prevalence of cerebellar hemangioblastoma ranges from 44% to 72%. Approximately 5-30% of all cerebellar hemangioblastomas are attributed to VHL disease^(6)^. Affected patients may present with ataxia, dysmetria, headache, diplopia, vertigo, or vomiting. Such symptoms occur because the cysts related to a hemangioblastoma grow much faster than does the primary tumor itself and have significant expansile effects^(1)^. The location in the cerebellar hemispheres may be related to the development of ataxia and dysmetria. Cerebellar lesions caused by VHL disease are close to the pial surface and are often cystic, with thin walls and eccentric solid components^(6)^. Computed tomography (CT) shows homogeneous cysts with well-defined walls and an eccentric mural nodule that, on unenhanced images, is isodense to the surrounding tissue, whereas it shows intense enhancement on contrast-enhanced images. On magnetic resonance imaging (MRI), hemangioblastomas have a cystic component with a signal that is hypointense on T1-weighted images and isointense or hyperintense on T2-weighted images (Figure 1). The solid component is classically characterized by facilitated diffusion and intense contrast enhancement^(1)^. Prominent flow voids related to tumor vascularization can often be seen. When a cerebellar hemangioblastoma is identified, it is important to actively look for other foci of enhancement throughout the neuraxis, given that the presence of other hemangioblastomas suggests VHL disease^(2,6)^. Hemangioblastomas present abundant vascularization due to increased expression of vascular endothelial growth factor^(7)^, which explains the elevated relative cerebral blood volume seen in perfusion sequences of these tumors (Figure 2).

## HEMANGIOBLASTOMA OF THE SPINAL CORD

**Figure 3:**
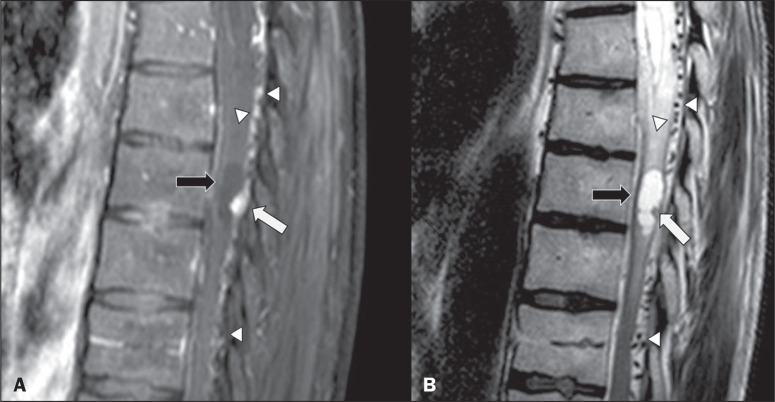
A 52-year-old male patient with VHL disease and spinal cord hemangioblastomas. Contrast-enhanced sagittal T1-weighted and T2-weighted MRI sequences (A and B, respectively), showing a solid/cystic intramedullary lesion at the level of the T9 vertebra. In A, note the eccentric solid nodule with enhancement (white arrow) and its cystic component (black arrow). On the T2-weighted sequence in B, note the hyperintense signal of the cystic component (black arrow) and the isointense signal of the posterior nodular component (white arrow), which corresponds to the eccentric solid nodule of the lesion. There are also superficial areas of enhancement in the spinal cord (in A), which correspond to flow voids (in B), which in turn correspond to dilated perimedullary veins (arrowheads).

Hemangioblastoma of the spinal cord is seen in 13-59% of patients with VHL disease. Although any segment of the spinal cord can be affected, hemangioblastomas are most common in the thoracic and cervical segments^(6)^. The predominant symptoms, which include hyperesthesia, weakness, ataxia, hyperreflexia, pain, incontinence, and even quadriplegia, are related to radiculopathy and myelopathy. Unenhanced CT scans show a nodule in the spinal cord that, on unenhanced images, is isodense to the surrounding tissue and, on contrast-enhanced images, shows intense enhancement. On MRI, a hemangioblastoma of the spinal cord tends to be hypointense on T1-weighted images and hyperintense on T2-weighted images, often with regional flow voids (Figure 3). These tumors also show intense enhancement on contrast-enhanced MRI scans and are accompanied by syringomyelia in 50-100% of cases^(1)^.

## METASTASIS

**Figure 4:**
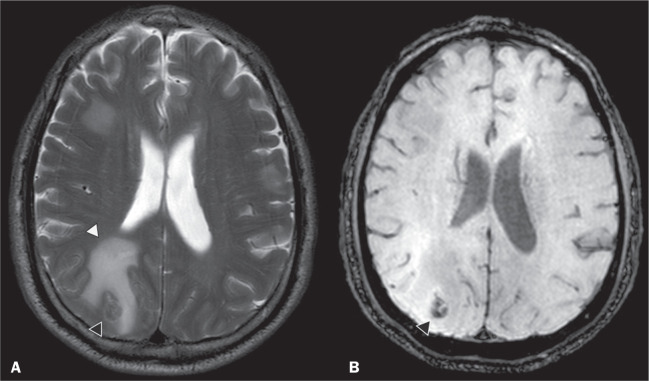
A 55-year-old male patient with VHL disease and a clear cell renal carcinoma with multiple metastases to the brain. T2-weighted MRI sequence showing a component with low signal intensity (black arrowhead in A) at the cortico-subcortical junction in the right parietal lobe, together with moderate perilesional edema (white arrowhead in A). In a susceptibility-weighted sequence (B), the cortico-subcortical lesion (arrowhead) shows a markedly hypointense signal (hemorrhage), characteristic of hemorrhagic brain metastasis (subsequently confirmed intraoperatively).

In patients with VHL disease, metastasis to the CNS most commonly originates from a clear cell renal carcinoma (Figure 4). Less commonly, metastases originate from a pheochromocytoma/paraganglioma or from a metastatic neuroendocrine tumor^(8)^.

## RETINAL HEMANGIOBLASTOMAS

**Figure 5:**
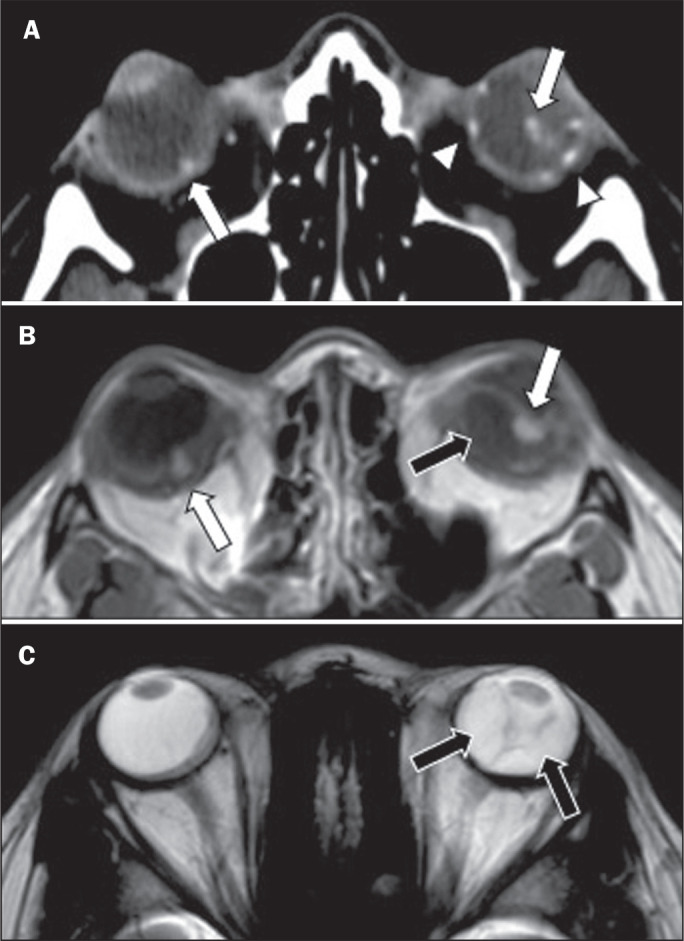
A 30-year-old female patient with VHL disease and retinal hemangioblastomas. Contrast-enhanced axial CT scan (A) showing nodules with enhancement throughout the right retina and in the posterior contour of the right globe and in the central region of the left globe (arrows), together with foci of calcification (arrowheads), consistent with retinal hemangioblastomas, in the left choroid region. Contrast-enhanced axial T1-weighted MRI sequence (B) showing nodular lesions with enhancement, one near the thickened posterior contour of the right globe, the other on the left near an anteriorized detached retina (white arrows), consistent with hemangioblastomas. Axial T2-weighted MRI sequence (C) showing the left retinal detachment in detail (arrows).

Retinal hemangioblastomas are common in VHL disease, being seen in up to 60% of patients. The mean age at presentation is 25 years, although it is estimated that up to 5% of cases occur in patients under 10 years of age^(3)^. Bilateral involvement is seen in up to 50% of cases. In up to 6% of patients, retinal hemangioblastomas cause complications such as macular exudation, exudative or tractional retinal detachment, vitreous hemorrhage, neovascular glaucoma, and amaurosis. Histopathological findings include fenestrated endothelial cells, pericytes, and lipid-rich foam cells in the stroma. The diagnosis is confirmed by ophthalmoscopy, which reveals a tumor with tortuous vessels and optic disc edema. Contrast-enhanced CT and MRI may reveal nodular retinal lesions with enhancement (Figure 5), with or without retinal detachment^(1)^.

## ENDOLYMPHATIC SAC TUMORS

**Figure 6:**
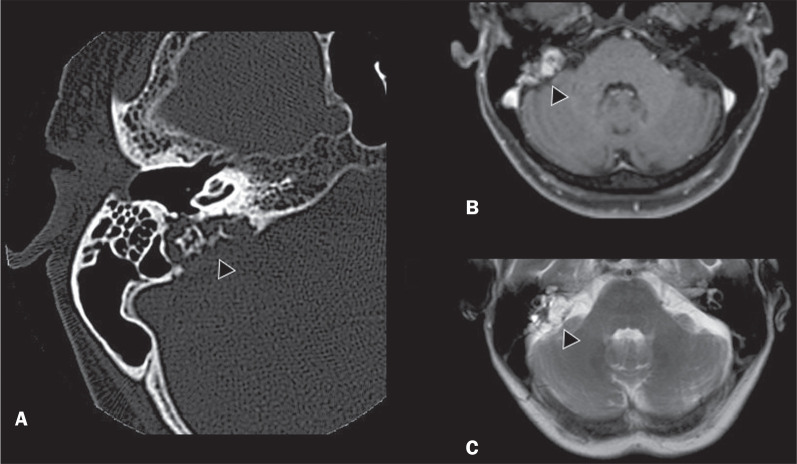
A 70-year-old female patient with VHL disease and an endolymphatic sac tumor. Axial CT scan with bone window settings (A), showing a lytic lesion (arrowhead) in the petrous portion of the right mastoid, with involvement of the internal auditory meatus and adjacent otic capsule. Contrast-enhanced T1-weighted MRI sequence (B) showing an expansile lesion centered on the posterior wall of the right petrous apex, characterized by heterogeneous enhancement (arrowhead). Axial T2-weighted MRI sequence (C) showing that the lesion signal was heterogeneously hyperintense (arrowhead).

Endolymphatic sac tumors occur in up to 15% of cases of VHL disease, the mean age at presentation being 22 years. Such tumors are bilateral in up to 30% of patients^(1,6)^. Endolymphatic sac tumors are highly vascularized papillary cystadenomas that grow in the posterior region of the petrous portion of the temporal bone^(3)^. These tumors arise from the vestibular aqueduct and are benign, although they are locally invasive and can erode adjacent structures, such as the semicircular canals and the cochlea. The symptoms are hearing loss, tinnitus, dizziness, and facial nerve palsy. A CT scan with bone window settings demonstrates a bone lesion with a moth-eaten appearance in the petrous portion of the temporal bone, with erosion of the vestibular aqueduct, semicircular canals, and cochlea. On contrast-enhanced images, an endolymphatic sac tumor presents intense enhancement. On MRI, such tumors show a signal that is hyperintense on T1-weighted images (denoting the presence of hemorrhagic/proteinaceous content) and heterogeneously hyperintense on T2-weighted images. Contrast-enhanced T1-weighted images demonstrate intense enhancement of solid tumor components^(1,6)^, as illustrated in Figure 6.

## FOLLOW-UP PROTOCOLS

All patients with VHL disease are predisposed to the development of benign or malignant lesions. Even if asymptomatic, such patients should be followed to detect new lesions and to monitor the progression of known lesions. Follow-up evaluations focus on hemangioblastomas (including retinal hemangioblastomas), endolymphatic sac tumors, pheochromocytomas, clear cell renal carcinomas, and pancreatic cystadenomas, as well as lesions of the epididymis and broad ligament of the uterus, and can be tailored to individual patient needs. Table 1 summarizes the current recommendations of the VHL Alliance Consensus^(9)^.

**Table 1:** Follow-up evaluations recommended for patients with VHL disease.*

Follow-up evaluation	Initial age	Frequency	Comments
Retinal assessment	< 1 year	Every 6-12 months	Annually, before age 30
Anamnesis and physical examination by a specialist	1 year	Yearly	
Blood pressure and heart rate	2 years	Yearly	
Determination of metanephrine levels	5 years	Yearly	Measurement in plasma preferred, but fractionated metanephrines may be measured in 24-h urine samples
MRI of the neuraxis (brain and spinal cord)	11 years	Every 2 years	Performed with and without contrast (no contrast during pregnancy); can be coordinated with MRI of the abdomen; thin slices in the posterior fossa and temporal bone; single MRI of the inner ear canal at 15 years of age
Audiometry	11 years	Every 2 years	
MRI of the abdomen	15 years	Every 2 years	Performed with and without contrast (no contrast during pregnancy); assess kidneys, pancreas, and adrenal glands; can be coordinated with MRI of the neuraxis

* Adapted from the VHL Family Alliance - VHL handbook^(9)^.
